# Clipping Noise Compensation for Overlapped Time Domain Multiplexing toward Low Peak-to-Average Power Ratio

**DOI:** 10.3390/s24051607

**Published:** 2024-03-01

**Authors:** Chongda Huang, Qianzhen Zhang, Xiaoyuan Li, Yue Xiao

**Affiliations:** 1 National Key Laboratory of Wireless Communications, University of Electronic Science and Technology of China, Chengdu 611731, China; 202121220119@std.uestc.edu.cn (C.H.); zhangqianzhen2022@163.com (Q.Z.); wshilixiaoyuan@163.com (X.L.); 2 School of Telecommunication Engineering, Xidian University, Xi’an 710126, China

**Keywords:** clipping distortion mitigation, Overlapped Time Domain Multiplexing (OvTDM), superposition coded modulation (SCM), peak-to-average power ratio (PAPR)

## Abstract

Overlapped Time Domain Multiplexing (OvTDM) is a high-rate transmission technology that employs the idea of superposition coded modulation (SCM) scheme for signal generation, aiming to achieve maximum channel capacity sharing. Meanwhile, it is also widely considered as a promising technique toward physical layer security. As a main drawback of such system, a high peak-to-average power ratio (PAPR) issue in this system, arising from multi-layer superposition, can be addressed through intentional clipping. However, the detection at the receiver side is vulnerable to nonlinear distortion caused by clipping, which can degrade the performance. To mitigate this distortion, this paper proposed an iterative scheme for estimating and partially canceling clipping distortion at the receiver. We managed to mitigate the impact of clipping noise as much as possible and minimize the cost of optimizing PAPR, thereby improving the transmission performance of OvTDM in the context of amplitude clipping.

## 1. Introduction

Overlapped Time Domain Multiplexing (OvTDM) [1,2] is an emerging modulation technique designed for high-rate transmission, rooted in superposition coded modulation (SCM) [3]. Positioned as a potential waveform for 6G wireless communications, OvTDM holds promise in specific scenarios [3,4,5]. Meanwhile, its application in physical layer security has been extensively explored in the current literature [6,7,8]. Despite its advantages in maximizing channel capacity sharing, OvTDM grapples with a notable peak-to-average power ratio (PAPR) problem, stemming from the multi-layer superposition in the modulation process.

To address this challenge, existing PAPR reduction techniques can be carefully considered which are broadly classified into two groups [9,10]. Specifically, the first group introduces redundancy to diminish the signal’s PAPR, such as partial transmit sequence (PTS) and coding [11,12]. However, this approach contradicts the high spectral efficiency pursuit of OvTDM. Meanwhile, the second group avoids additional redundancy, exemplified by the clipping technique, which introduces extra interference to the signal [13,14]. Unfortunately, when combined with the second group of techniques such as clipping, OvTDM signals will suffer from nonlinear distortion, leading to the considerable degradation of transmission performance.

To counter the distortion induced by clipping, researchers have explored a class of iterative recovery methods that achieve the estimation and partial cancellation of clipping distortion at the receiver [15,16,17,18,19,20,21,22]. Specifically, the common principle underlying these iterative detection schemes is to continuously and effectively mitigate clipping noise through iterative detection, thus obtaining a more accurate estimation of the original signal. Therefore, the iterative detection process involves the straightforward reconstruction of the clipping noise as well as employing a soft iterative compensation approach. In general, the existing literature characterizes clipping noise in various ways, each offering different performance enhancements and implementation complexities [23,24].

In this paper, the primary focus is on addressing the high PAPR challenge within the OvTDM system. To tackle this issue effectively, based on the basic framework of iterative detection, we propose a soft iterative compensation scheme carefully designed under a well-defined mathematical model characterizing the inherent clipping noise in such system. These developed compensation strategies are capable of systematically reducing the PAPR of OvTDM signals, thereby enhancing the overall performance and efficiency of the OvTDM system.

The remainder of the paper is organized as follows: Section 2 provides a description of the system and discusses the challenges posed by clipping distortion. Section 3 details the soft iterative detection under three different clipping noise models while describing the iterative detection of directly reconstructed clipping noise. Simulation results and comparative analysis are presented in Section 4. Finally, Section 5 concludes the paper with a summary of the findings and directions for future research.

## 2. Transmitter and Receiver of OvTDM Systems

In general, OvTDM is a time multiplexing technique aiming at maximizing channel capacity sharing, so it differs fundamentally from the traditional time multiplexing technique (TDM). Instead of providing guard intervals for each signal, OvTDM deliberately induces overlapping by considering it as a constraint relationship. The more overlapping occurs, the higher the coding gain, which implies a longer coding constraint length. By superimposing waveforms, it is possible to transmit more symbol waveforms within the same time, thereby providing higher spectral efficiency compared to conventional modulation techniques.

**Figure 1:**
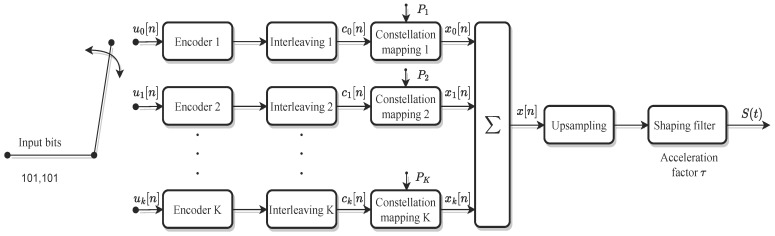
Block diagram of the transmitter for OvTDM.

The structure of the transmitter side of the system is shown in Figure 1. The source bits $U={\left[u_{0},u_{1},u_{2},\ldots ,u_{M}-1\right]}^{T}$, $u_{n}\in \left\{0,1\right\}$ are first transformed into K-layer signals through a serial-parallel transformation, and then coded and modulated, respectively, while $c_{k}$ is the codeword stream after interleaving. In order to reduce the complexity of both the transmitter and receiver, the same coding and modulation methods are used for each layer. While modulating the signal, the power is allocated to each layer of symbols as $P_{1},P_{2},\ldots ,P_{K}$. The symbol stream after mapping to K-layer can be denoted by ${\left[x_{1},x_{2},\ldots ,x_{K-1}\right]}^{T}$, and the K-layer signals are superimposed on the transmitter to produce the final symbol stream. Therefore, $x_{k}\left[n\right]$ after power allocation can be expressed as

(1) $$x\left[n\right]=\sum_{k=1}^{K}\sqrt{P_{k}T}x_{k}\left[n\right].$$

After overlapping, the symbol stream x[n] is up-sampled and pulse-formed with sampling intervals *T*, which is orthogonal during the symbol period. During the shaping-filter process, if a further increase in spectral efficiency is required, a smaller transmission interval can be used to send the signals as τT, (τ≤1). Thus, the final OvTDM signal can be expressed as follows:

(2) $$\begin{array}{ccc} S(t) & = & \sum_{n=0}^{N-1}x[n]h(t-n\tau T)\\ & = & \sum_{k=0}^{K-1}\sum_{n=0}^{N-1}x_{k}\left[n\right]h\left(t-n\tau T\right), \end{array}$$

where *N* is the number of symbols in each layer, the default binary phase shift keying (BPSK) modulation is applied, and h(t) is the energy-normalized pulse-shaping filter waveform. And, the accelerating factor, denoted by τ, plays a crucial role in shortening the sampling interval of the sinc function. In signal processing, the sinc function is commonly used for sampling and reconstruction. The introduction of the accelerating factor τ allows for a more rapid adjustment of the sampling intervals, facilitating quicker and more efficient signal processing.

**Figure 2:**
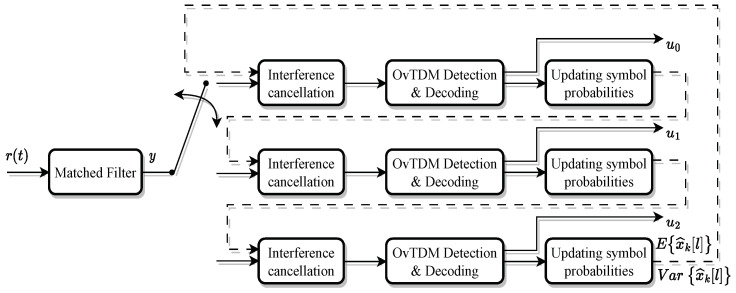
Block diagram of the receiver using SIC detection for OvTDM.

Assuming that the signal passes through a Gaussian white noise channel, the received signal r(t) can be expressed as

(3) $$r\left(t\right)=\sum_{n=0}^{N-1}x_{n}h\left(t-n\tau T\right)+\eta \left(t\right),$$

where η(t) is the Gaussian white noise in the channel. The receiver model of OvTDM is shown in Figure 2, and the received signal after matched filtering and sampling can be expressed as

(4) $$y[n]=\int_{-\infty }^{+\infty }r\left(t\right)h^{*}(t-n\tau T)dt,$$

where $h^{*}(t)$ is the conjugate of the pulse shaping function h(t) used at the transmitter.

The received signal *y*, after the matched filtering and sampling process, *y* is obtained as a sufficient statistic, expressed as

(5) y=Gx+η,

where η is the sampled value of the color noise and *G* is the Toplitz matrix as

(6) $$\begin{array}{c} G=\left(\begin{array}{ccccccccc} 1 & g_{-1} & \ldots & g_{-L_{ISI}} & 0 & 0 & 0 & 0 & \ldots \\ g_{1} & 1 & g_{-1} & ... & g_{-L_{ISI}} & 0 & 0 & 0 & \ldots \\ \vdots & \ddots & \ddots & \ddots & & \ddots & & \ddots & \\ g_{L_{ISI}} & \ldots & g_{1} & 1 & g_{-1} & \ldots & g_{-L_{ISI}} & 0 & \ldots \\ 0 & g_{L_{ISI}} & \ldots & g_{1} & 1 & g_{-1} & \ldots & g_{-L_{ISI}} & \ldots \\ \vdots & & \ddots & & \ddots & \ddots & \ddots & & \ddots \end{array}\right) \end{array}$$

where $g_{n}$ is the interference coefficient of the *n*-th symbol adjacent to the current symbol on the current symbol, and $L_{ISI}$ is the number of unilateral interference symbols. Expanding the matrix form gives

(7) $$\begin{array}{ccc} y[n] & = & \sum_{m=0}^{N-1}x\left[m\right]g_{n-m}+\eta \left[n\right]\\ & = & \sum_{k=0}^{J-1}\sum_{m=0}^{N-1}x_{k}\left[m\right]g_{n-m}+\eta \left[n\right], \end{array}$$

where $g_{n}$ is the interference coefficient of the *n*-th symbol adjacent to the current symbol. From the above equation, it can be seen that the sampled value at the current moment will be affected not only by the interference between symbols in the same layer, but also by the interference in other layers as well as the color noise.

For the OvTDM system with SCM, at the receiver side, the Forney model [25] adds a whitening filter on the receiving end, which transforms color noise into white noise. Hence, the one-by-one interference cancellation algorithm will be used to process the interference of the other layers first. Meanwhile, for each layer of signal, the M-BCJR detection algorithm based on the Forney model will be used to deal with the effect of the inter-symbol interference and the channel noise [26,27]. The whole process of receiving end detection can be briefly described as follows.

(i) The detection at the receiver side starts from the layer with the highest transmit power, where $P_{0},P_{1},\ldots ,P_{K-1}$ decreases sequentially. First, initialize the symbol probability $P({\hat{x}}_{k}\left[l\right])$ for each layer of symbols as an equal probability distribution, and then calculate the mean $E\left\{{\hat{x}}_{k}\left[l\right]\right\}$ and variance $\mathrm{Var}\{{\hat{x}}_{k}\left[l\right]\}$ of the symbols at each moment of each layer based on the probability, with k=0.
(ii) Let the current decoding layer be the k-th layer, and firstly calculate the mean magnitude $\mu_{k}$ and variance $\sigma_{k}^{2}$ of the interference values caused by other layers to the current layer.
(iii) The input $y_{k}$ to the *k*-th layer OvTDM signal detector is obtained by subtracting $\mu_{k}$ from the sufficient statistic *y* obtained after matched filtering and down-sampling.
(iv) Detection of $y_{k}$ using the M-BCJR algorithm.
(v) The symbol probability obtained at the end of M-BCJR detection is fed into the decoder, the detector and decoder are iterated a number of times to obtain the estimated bit sequence and the corresponding probability information for the current layer, and the obtained bit probability is re-mapped into the symbol probability to recalculate the symbol mean-variance. Then, update $E\{{\hat{x}}_{k}\left[l\right]\}$ and $\mathrm{Var}\{{\hat{x}}_{k}\left[l\right]\}$. When k≠K−1, let k=k+1, restart the detection of the next layer of signals from step (ii), otherwise jump to step (vi).
(vi) When k=K+1, it means that each layer of the signal is detected. At this time, $E\{{\hat{x}}_{k}\left[l\right]\}$ and $\mathrm{Var}\{{\hat{x}}_{k}\left[l\right]\}$ of each layer have been updated once. The updated $E\{{\hat{x}}_{k}\left[l\right]\}$ and $\mathrm{Var}\{{\hat{x}}_{k}\left[l\right]\}$ can be used to restart the detection from the first layer by ordering k=0, and restarting the detection from step (ii). At this point, $E\{{\hat{x}}_{k}\left[l\right]\}$ and $\mathrm{Var}\{{\hat{x}}_{k}\left[l\right]\}$ are much more accurate compared to the homogeneous initialization with the first step, and thus the second overall iteration will provide a better performance than the first one. As results, a number of iterations of detection can achieve better performance.

## 3. PAPR Reduction with Clipping and Modified Iterative Detection Scheme

Consider the OvTDM transmitter scheme depicted in Figure 1, where the signal is clipped before transmission in order to reduce the PAPR. The clipping function c(x) can be written as

(8) $$c\left(x\right)=\left\{\begin{array}{c} x\ldots \ldots \ldots (\left|x\right|\leq A)\\ \frac{Ax}{\left|x\right|}\ldots \ldots \ldots \left(\left|x\right|\geq A\right), \end{array}\right.$$

where *x* represents the input signal. The clipping threshold, denoted by *A*, serves as a limit amplitude threshold in signal processing. This threshold defines the maximum amplitude value beyond which a signal will undergo amplitude clipping. In other words, when the signal’s amplitude surpasses the specified threshold *A*, the signal is restricted or “clipped” to the predefined maximum value.

For convenience, the parameter clipping ratio (CR) is usually used as an alternative representation of the clipping threshold with the expression as

(9) $$CR=\frac{A^{2}}{E\left[{\left|s\left(t\right)\right|}^{2}\right]}.$$

Note that clipping is a nonlinear process that leads to severe in-band interference and out-of-band noise, so as to degrade the performance of the entire system. Therefore, this paper focuses on the in-band signal effects to minimize the BER performance loss at the receiver side.

The assumptions in this paper are firstly described as follows.

- The signal processing at the receiver side uses the Forney model, i.e., after down-sampling the signal passes through a whitening filter. Thus, the total noise can be written as an additive Gaussian white noise.
- Since multiple layers are superimposed, the distribution of the signal in the time domain should asymptotically obey a Gaussian distribution. Here, it is assumed that the signal time domain obeys Gaussian distribution. Also, since BPSK is used as the baseband modulation, the transmitted signal is a real signal and its amplitude in the time domain obeys the following distribution as
  (10) $$f\left(x\right)=\frac{1}{\sqrt{2\pi }\sigma_{x}}e^{-\frac{x^{2}}{2{\sigma_{x}}^{2}}}.$$

### 3.1. Iterative Soft Compensation

Here, it is assumed that the signal vector before clipping is $x=\{x_{1},x_{2},\ldots ,x_{N}\}$. According to the existing literature [3,18,19,24], the nonlinear distortion introduced by c(x) can be separated into two parts as

(11) c(x)=αx+d,

where d is the clipping noise vector, usually considered to be Gaussian noise uncorrelated with the signal. And, α is an accelerating factor which is calculated as

(12) $$\alpha =\frac{E\left[x^{H}c\left(x\right)\right]}{E\left[{∥x∥}^{2}\right]}.$$

Then, the signal at the receiving end can be expressed as

(13) y=αx+d+n,

where n is the additive Gaussian noise of the channel. It is then necessary to consider the probability distribution model of d. In general, d is usually considered a zero-mean Gaussian noise, then at this point, d can be combined with n into a total Gaussian noise $w_{1}=n+d$ with the total noise variance $\sigma_{w_{1}}^{2}=\sigma_{n}^{2}+\sigma_{d}^{2}$. Therefore, only a correction for the estimated noise power at the receiver side is required under this assumption, while the variance of d can be expressed as

(14) $${\sigma_{d}}^{2}=\left(1-\alpha^{2}\right){\sigma_{x}}^{2},$$

where the transmit power ${\sigma_{x}}^{2}$ is usually normalized to 1.

Meanwhile, another way of estimating d is to model it as a Gaussian variable with a non-zero mean. Thus, the parameters d are constantly updated through iterative testing to obtain a more accurate noise estimate to improve the decoder performance. So the signal to be demodulated at the receiver side can be written as

(15) $$y-\bar{d}=\alpha x+\left(d-\bar{d}\right)+n=\alpha x+w_{2},$$

where $w_{2}=\left(d-\bar{d}\right)+n$ and $\bar{d}$ is the mean value of d. In the previous section, we assumed that the signal time domain is Gaussian distributed, then x can be completely described by the mean E[x] and variance V[x]. Thus, the mean value of d can be calculated by the following equation

(16) $$\begin{array}{ccc} \bar{d}\left[n\right] & = & E[c(x[n])-\alpha x[n]]\\ & = & \int \frac{c(x)-\alpha x}{\pi V[x[n]]}e^{-\frac{|x-E\left[x\left[n\right]\right]{|}^{2}}{\;V[x[n]]}}dx. \end{array}$$

Correspondingly, the variance of d can be expressed as

(17) $$\begin{array}{ccc} V[d[n]] & = & E\left[|d\left[n\right]-\bar{d}\left[n\right]{|}^{2}\right]\\ & = & \int \frac{|c\left(x\right)-\alpha x-\bar{d}\left[n\right]{|}^{2}}{\pi V[x[n]]}e^{-\frac{|x-E\left[x\left[n\right]\right]{|}^{2}}{\;V[x[n]]}}dx. \end{array}$$

Then, the total noise variance can be expressed as

(18) $$\begin{array}{ccc} \sigma_{w_{2}}^{2} & = & \sigma_{n}^{2}+\sigma_{d}^{2}\\ & = & \sigma_{n}^{2}+\sum V[d[n]], \end{array}$$

and this corrected noise variance is used in the log-likelihood ratio calculation of the decoder. In summary, the estimation algorithm for clipping noise can be expressed as follows.

(i) The first round of SIC detection is performed by initializing $\bar{d}$ to 0. After obtaining the updated $E\{{\hat{x}}_{k}\left[l\right]\}$ and $\mathrm{Var}\{{\hat{x}}_{k}\left[l\right]\}$ for each layer, they are superimposed in the same way as in the modulation process. If τ≠1, then the inter-symbol interference coefficient $g_{n}$ must also be taken into calculation. Thus, the E[x] and V[x] of the overall signal can be estimated.
(ii) Calculate $\bar{d}$ via (16) and let $\hat{y}=y-\bar{d}$. Calculate $\sigma_{d}^{2}$ via $\bar{d}$ and (17) and modify the total noise variance $\sigma_{w_{2}}^{2}$ for the next iteration.

**Figure 3:**
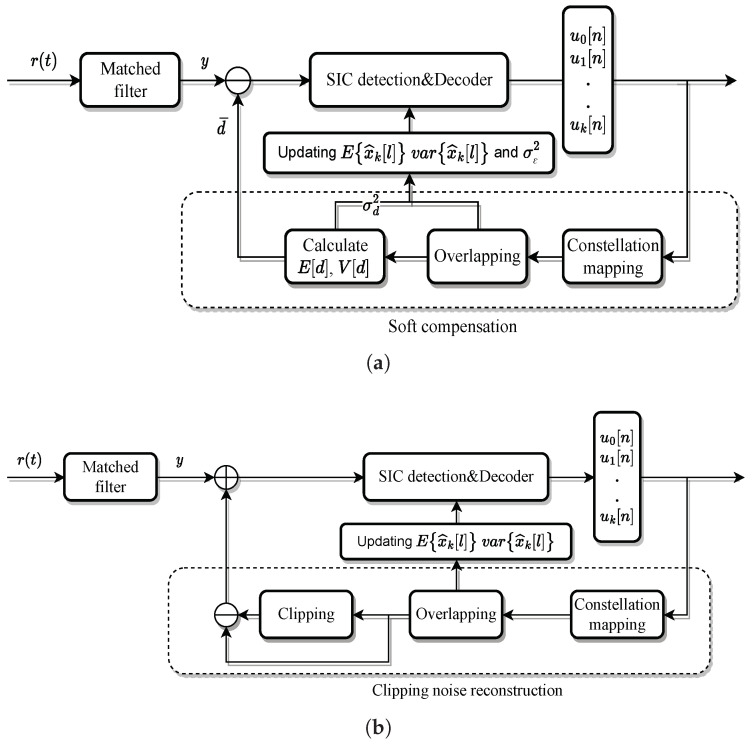
Block diagram of the iterative detection algorithm for clipped OvTDM. (**a**) Block diagram of iterative soft compensation algorithm for OvTDM. (**b**) Block diagram of clipping noise reconstruction for OvTDM.

In general, such a clipping noise estimation method is blended with the original iterative detection process, so only a few estimation calculations are added to each iteration of the process, which does not increase the complexity much, as shown in Figure 3a.

### 3.2. Clipping Noise Reconstruction

For dealing with the clipping noise, another approach is to directly reconstruct the clipping noise in the iterative detection and correct the received signal by the reconstructed clipping noise, as shown in Figure 3b.

Here again, the signal after clipping is modeled as (13). In the iterative detection process at the receiver side, the following algorithm is introduced.

(i) The first iteration yields a preliminary demodulation result $\hat{U}={\left[\hat{u_{0}},\hat{u_{1}},\hat{u_{2}},\ldots ,\hat{u_{M}-1}\right]}^{T}$, with $u_{n}\in \left\{0,1\right\}$ by SIC detection and decoding.
(ii) Perform the same modulation process on the detection results as on the transmitter to reconstruct the transmit signal estimate $\hat{x}$.
(iii) Reconstruct the clipping noise according to
  (19) $$\hat{d}=c\left(\hat{x}\right)-\alpha \hat{x},$$
  which is then subtracted from the received signal as $\hat{y}=y-\hat{d}$, and replace y by $\hat{y}$ in the next round of iterative detection.

With such above-mentioned iterative detection scheme, the clipping noise is estimated more accurately with multiple iterations, resulting in more accurate decoding results.

To evaluate the complexity, we assume that the signal length is *N*. The proposed scheme involves several operations in each iteration, including *N* times numerical integration, one *N*-point inverse fast Fourier transform (IFFT), one *N*-point fast Fourier transform (FFT), and one *N*-dimensional vector addition.

The time complexity of the *N* times numerical integration is O(N). The *N*-point IFFT and *N*-point FFT have a time complexity of O(Nlog(N)) each. Meanwhile, the *N*-dimensional vector addition takes O(N) time. Therefore, the total time complexity is given by O(Nlog(N))+O(N)+O(N), which simplifies to O(Nlog(N)).

In each iteration of the existing serial interference cancellation framework, the introduced iterative approach includes a corrective measure involving amplitude-limited noise. As a result, the computational complexity of the modified scheme shows a moderate increase when compared to the original approach. This indicates a smooth integration of the proposed solution with the existing framework, resulting in a negligible rise in computational overhead. Essentially, the incremental complexity introduced by the proposed scheme is small, affirming its compatibility and efficiency within the established paradigm.

## 4. Simulation Results

In this section, the simulation results in this paper are given to demonstrate the effectiveness of the proposed method. The error correction code selected is Turbo code, with the coding rate of 1/3 and the number of overlapping layers K=3. At the receiver side, the number of iterations for SIC detection is 3. Other specific simulation parameters are listed in Table 1.

**Figure 4:**
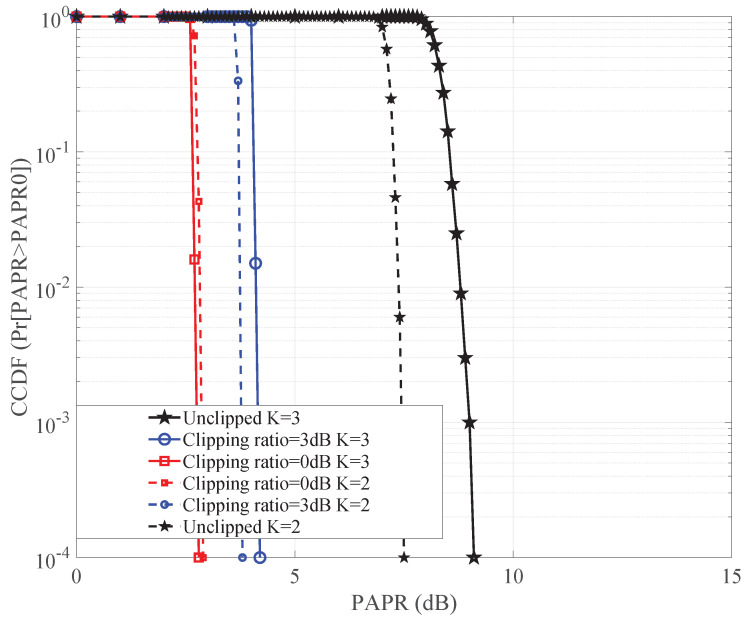
PAPR of the signal with different clipping ratios.

By examining the details presented in Figure 4, we observe a significant difference in PAPR between the original OvTDM-modulated signal and the signal undergoing the clipping procedure. This result illustrates the impact of clipping on the signal’s peak power, emphasizing the effectiveness of this technique in managing the PAPR.

**Figure 5:**
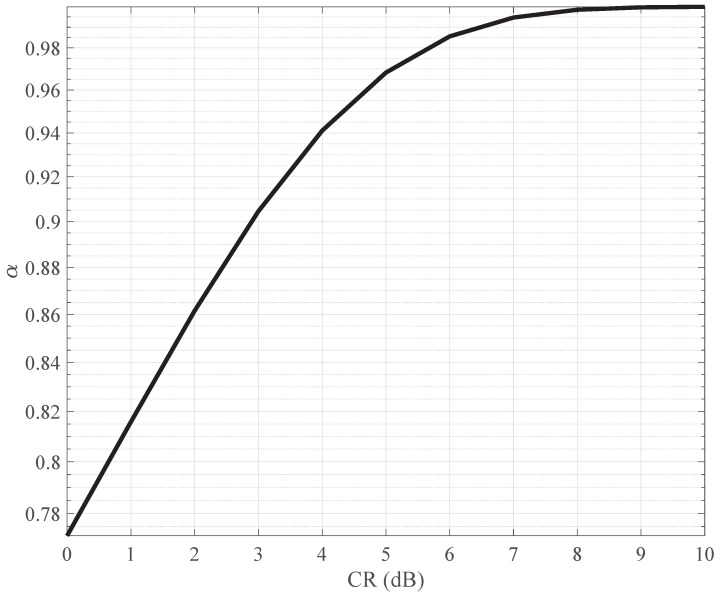
Values of α under different clipping ratios.

Moving on to Figure 5, we delve into the intricate relationship between the signal accelerating factor α and various clipping ratios. As the clipping ratio increases, indicating a more aggressive clipping process, the PAPR of the signal notably decreases. This reduction is promising, suggesting improved signal quality in terms of decreased peak power. However, it is essential to note that this reduction in PAPR comes at the cost of a diminishing α value.

The declining α value indicates an increase in signal interference, highlighting a trade-off between PAPR reduction and the interference introduced by the clipping process. Achieving an optimal balance between PAPR reduction and acceptable interference levels is crucial for efficient signal transmission and reception in the given system.

**Figure 6:**
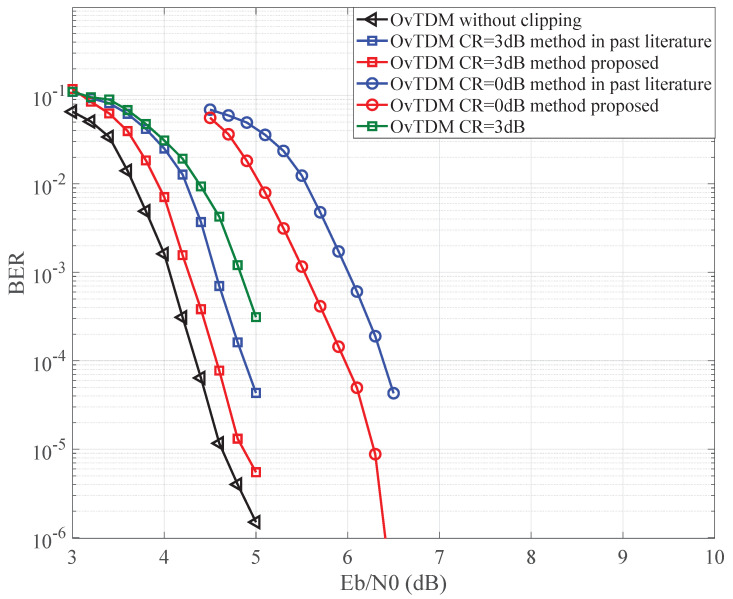
BER performance comparison when K=3 under Gaussian channel with method in [18].

**Figure 7:**
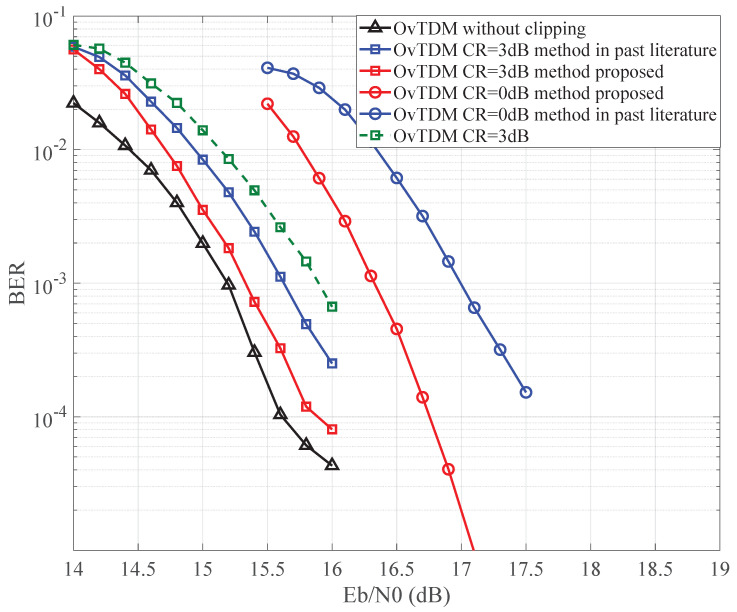
BER performance comparison when K=3 under TDL-C channel with method in [18].

Figure 6 and Figure 7 offer a comprehensive view of the bit error rate (BER) performance for OvTDM, showcasing the impact of different iterative detection methods. Graphically illustrating the gain observed in our proposed iterative detection scheme with CR set at 0 dB and 3 dB in Figure 6 and Figure 7, the application of amplitude clipping does lead to a considerable deterioration in BER performance. Nonetheless, our devised methodology effectively mitigates this decline.

Through the synergistic implementation of soft iteration and noise reconstruction, we have observed a noteworthy performance improvement of approximately 0.4–0.6 dB, showcasing the compensatory prowess of the proposed approach. The discernible trend reveals the promising potential of the proposed modified iterative detection algorithm, underscoring its effectiveness in significantly enhancing the BER performance when compared to the original detection method. Furthermore, the soft compensation iterative detection approach exhibits a marginal advantage over the direct reconstruction of clipping noise iterative detection. While there is a nuanced difference, overall, both approaches demonstrate comparable performance.

The iterative strategy implemented on the receiver side, from a cornerstone of this study, gives an accurate estimation and reconstruction of the clipping noise during signal detection of OvTDM. This reduction is pivotal for ensuring reliable transmission in high-speed systems, effectively balancing the imperative of PAPR suppression with the need for a resilient and dependable communication channel. Thus, when applying OvTDM, the quest for efficient and robust high-speed data transmission with controlled PAPR is made attainable.

## 5. Conclusions

In summary, this study considered the issue of PAPR reduction for OvTDM systems in the context of clipping. We demonstrated that, while deliberate clipping effectively addresses concerns related to high PAPR, it simultaneously introduces considerable nonlinear distortion. Therefore, this contribution proposed a class of receiver-side iterative recovery schemes for mitigating the impact of clipping. Specifically, we focus on achieving accurate clipping noise estimation through iterative techniques, ultimately enhancing the decoding outcomes. We exhibited that the proposed technique can significantly improve the reliability and efficiency of OvTDM, particularly when dealing with distortions induced by clipping. Since such a receiver-side strategy provides practical approaches to counteract nonlinear distortion, the effectiveness of OvTDM is further improved in high-capacity channel sharing. In future works, we will focus on quantifying the effect of clipping on OvTDM with coding and iterative processing in different system configurations. We look forward to further exploring these topics and contributing to the advancement of the field.

## Figures and Tables

**Table 1:** Parameters of the OvTDM system.

Parameter	Value
Modulation scheme	BPSK
Number of layers	3
Data length	1000
Encoding/rate	Turbo 1/3
Symbols per layer	3010
FTN Accelerating factor (τ)	0.8

## Data Availability

Data are contained within the article.
